# Ceftazidime/Avibactam Resistance in Carbapenemase-Producing *Klebsiella pneumoniae*

**DOI:** 10.3201/eid2911.230830

**Published:** 2023-11

**Authors:** Qiaozhen Cui, Chen Wang, Qichen Wang, Juanxiu Qin, Min Li, Baixing Ding, Zhen Shen

**Affiliations:** Shanxi Provincial People's Hospital, Taiyuan, China (Q. Cui);; Renji Hospital at Shanghai Jiao Tong University School of Medicine, Shanghai, China (C. Wang, Q. Wang, J. Qin, M. Li, Z. Shen);; Huashan Hospital at Fudan University, Shanghai (B. Ding);; Key Laboratory of Clinical Pharmacology of Antibiotics, Ministry of Health, Shanghai (B. Ding)

## Abstract

We identified a novel ceftazidime/avibactam resistance mechanism in sequence type 11 *Klebsiella pneumoniae* carbapenemase 2–producing *K. pneumoniae*. Plasmid recombination and chromosomal integration formed a novel virulence plasmid and provided an additional promoter for *bla*_SHV-12_, leading to *bla*_SHV-12_ overexpression and ceftazidime/avibactam resistance. Genetic rearrangement contributed to convergence of hypervirulence and ceftazidime/avibactam resistance.

Emergence and global dissemination of carbapenem-resistant *Klebsiella pneumoniae* pose therapeutic challenges to public health (*1*). The most crucial cause of carbapenem resistance in *K. pneumoniae* is carbapenemase production; thus, the novel β-lactamase inhibitor ceftazidime/avibactam (CAZ/AVI) provides an antimicrobial strategy (*1*–*3*). However, its increasing use raises resistance concerns. According to the China Antimicrobial Surveillance Network (http://www.chinets.com/Data/AntibioticdrugFast), 9.9% of *K. pneumoniae* carbapenemase (KPC) 2–producing *K. pneumoniae* (KPC-KP) displayed CAZ/AVI resistance (*4*). β-lactamase amino acid substitutions are the dominant mechanisms that lead to CAZ/AVI resistance (*5*). Mutations in class A β-lactamases, especially KPCs, have been reported (*5*). Substitutions in KPCs could improve ceftazidime affinity or reduce avibactam inhibition (*5*). We report a novel CAZ/AVI resistance mechanism in epidemic sequence type (ST) 11 KPC-KP. All study procedures involving human participants and animals were in accordance with the ethics standards of the Institutional Review Board Ethics Committee of Shanxi Provincial People's Hospital; this type of retrospective study did not require formal consent.

In 2021, a 62-year-old man was transferred from another hospital to a teaching hospital in Shanxi Province, China. Before transfer, a blood culture indicated CAZ/AVI–susceptible carbapenem-resistant *K. pneumoniae*. The patient received 1 week of CAZ/AVI therapy before transfer and another week of CAZ/AVI therapy after admission. In addition to the bloodstream infection, severe pneumoniae, multiple duodenal ulcers, and gastrointestinal hemorrhage developed. Two weeks after CAZ/AVI withdrawal, we isolated KP0714, which was resistant to all β-lactams tested but susceptible to tigecycline and polymyxin B (Appendix Table 1). We generated the KP0714 complete genome by using the combination of Illumina and PacBio RS sequencing (Appendix Table 2), and it belonged to ST11. The resistance plasmid pKPC-KP0714 carries *bla*_KPC-2_ and several other resistance genes, including *bla*_TEM-1_, *rmtB*, and *fosA3* (Appendix Table 2), and KPC-2 S130A substitution was constructed in situ. KP0714 and the KPC-2 S130A mutant displayed the same MICs for CAZ/AVI, suggesting that *bla*_KPC-2_ was not involved in CAZ/AVI resistance (Appendix Table 1).

KP0714 possessed a novel IncFIB(K)-type virulence plasmid pVir-KP0714, encoding siderophore aerobactin (*iucABCDiutA*) and capsular polysaccharide regulator RmpA2. pVir-KP0714 was 99.97% identical to reference plasmid pOXA1_020030 (GenBank accession no. CP028791) from *K. pneumoniae* strain WCHKP020030 at 74% coverage. Both ends of pVir-KP0714 were absent from pOXA1_020030 but were highly homologous to another plasmid, pLAP2_020030 (GenBank accession no. CP028792), from WCHKP020030 (Figure 1; Appendix Figure 1). Multiple mobile genetic elements on these plasmids suggested that pVir-KP0714 was generated through genetic recombination between pOXA1_020030 and pLAP2_020030, which not only formed a novel virulence plasmid but also contributed to chromosomal integration of a 45-kb plasmid fragment from pLAP2_020030 (Figure 1; Appendix Figure 1). The 45-kb fragment that had not been integrated into pVir-KP0714 was divided into the upstream 30-kb fragment and a 15-kb genetic context containing *bla*_SHV-12_, which were independently inserted into the chromosome. The 15-kb genetic context containing *bla*_SHV-12_ was flanked by several IS26 insertion sequences and harbored 3 other resistance genes, *bla*_LAP-2_, *qnrS1*, and *aph(3′)-Ia*, which exhibited 100% identity and 100% query coverage with the reference plasmid pLAP2_020030 (Figure 2; Appendix Figure 2).

**Figure 1:**
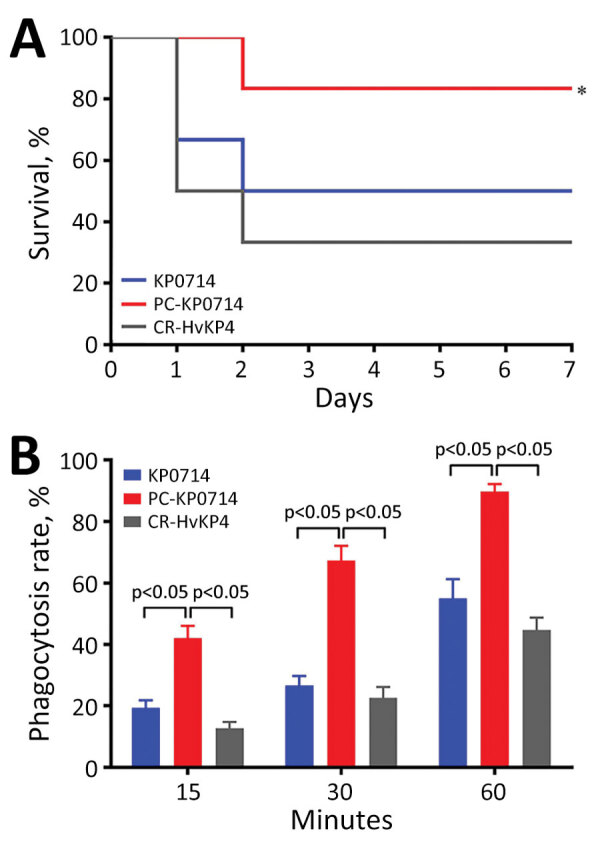
Linear alignment of plasmid pVir-KP0714 and virulence potential determination of *Klebsiella.* *pneumoniae* isolate KP0714 in study of ceftazidime/avibactam resistance in carbapenemase-producing *K. pneumoniae*. A) Virulence potential determination of KP0714 and pVir-KP0714–curing mutant (PC-KP0714) in a mouse infection model. Sequence type 11 carbapenem-resistant hypervirulent *K. pneumoniae* strain CR-HvKP4 was used as a hypervirulence control. Bacterial suspensions in the logarithmic growth phase were diluted in sterile phosphate-buffered saline to 10^7^ CFU/mL. Six female BALB/c mice were used as a sample population for each isolate. BALB/c mice were infected intraperitoneally with 0.1 mL of the diluted bacterial suspension. Clinical signs and mortality rates were noted for 7 days. *p<0.05 when compared with PC-KP0714. B) Human neutrophil assays of KP0714. Error bars indicate SDs. p values were computed by 1-way analysis of variance with Bonferroni correction.

**Figure 2:**
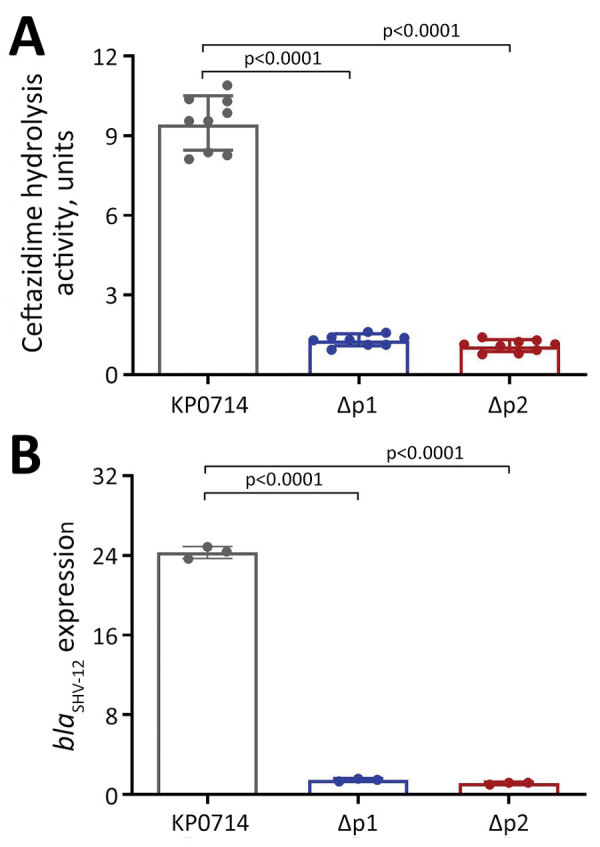
Overexpression of *bla*_SHV-12_ contributing to ceftazidime/avibactam resistance in *Klebsiella.* *pneumoniae* isolate KP0714 in study of ceftazidime/avibactam resistance in carbapenemase-producing *K. pneumoniae*. A) Relative *bla*_SHV-12_ expression level. B) Ceftazidime hydrolysis activity of different *bla*_SHV-12_ promoter deletion mutants. One unit of enzyme activity was defined as the amount of enzyme that hydrolyzed 1 nmol of substrate per min. Error bars indicate SDs. p values were computed by 1-way analysis of variance with Bonferroni correction.

However, we observed substantial structural changes in this chromosomal insertion fragment compared with pLAP2_020030 (Figure 2; Appendix Figure 2). The reversion and rearrangement of IS26-*aph(3′)-Ia* provided an addition promoter P2 for *bla*_SHV-12_ (Figure 2; Appendix Figure 2). To determine the role of promoter P2 in CAZ/AVI resistance, we deleted P2 and the original promoter P1 of *bla*_SHV-12_ by using a pConj working vector-based genetic engineering approach (*6*). Deletion of P1 or P2 could completely restore KP0714 susceptibility to CAZ/AVI; CAZ/AVI MICs were 2 and 1 μg/mL, respectively (Appendix Table 1). The relative expression of *bla*_SHV-12_ in KP0714 was ≈20-fold higher than in ΔP1 and ΔP2 mutants (Figure 2; Appendix Figure 2). Similarly, the hydrolysis activity of ceftazidime in KP0714 was significantly higher than that of ΔP1 and ΔP2 mutants (p<0.0001). Those results demonstrated that CAZ/AVI resistance in KP0714 was attributed to overexpression of *bla*_SHV-12_ resulting from an additional promoter, and the original promoter P1 was also necessary for the biological function of P2.

Because a novel virulence plasmid pVir-KP0714 was formed through plasmid recombination, we determined the virulence potential of KP0714 by using a mouse infection model and human neutrophil phagocytosis assay (*7*). As the hypervirulence control, we used the previously reported ST11 carbapenem-resistant hypervirulent *K. pneumoniae* strain CR-HvKP4 (*8*). We found no statistical difference regarding mouse survival and neutrophil phagocytosis between KP0714 and CR-HvKP4 (Figure 1; Appendix Figure 2), suggesting convergence of hypervirulence and CAZ/AVI resistance in KP0714. In contrast, mouse survival rates were significantly higher and human neutrophil phagocytosis rates were significantly lower for KP0714 and CR-HvKP4 at each time point when compared with virulence plasmid pVir-KP0714-curing KP0714 (PC-KP0714), demonstrating that KP0714 hypervirulence was attributed to acquisition of virulence plasmid pVir-KP0714.

In conclusion, KP0714 high-level resistance to carbapenems and CAZ/AVI, compensating for decreased carbapenem hydrolyzation activity of KPC variants (*5*,*9*), highlights a novel evolution pathway for development of CAZ/AVI resistance in epidemic ST11 KPC-KP, posing a threat to clinical antimicrobial therapy. Emerging CAZ/AVI-resistant and hypervirulent ST11 KPC-KP might be continuously evolving and warrants prospective monitoring.

Appendix Additional results for study of genetic rearrangement and ceftazidime/avibactam resistance in hypervirulent *Klebsiella pneumoniae*.
