# Supplementary material for: Ceftazidime/Avibactam Resistance in Carbapenemase-Producing Klebsiella pneumoniae
Source: Emerg Infect Dis. 2023 Nov;29(11):2398–400. doi: 10.3201/eid2911.230830 (PMC10617341; doi:10.3201/eid2911.230830)
Supplement: Appendix — Additional results for study of genetic rearrangement and ceftazidime/avibactam resistance in hypervirulent Klebsiella pneumoniae. [file 23-0830-Techapp-s1.pdf]

Article DOI: https://doi.org/10.3201/eid2911.230830

EID cannot ensure accessibility for supplementary materials supplied by authors. Readers who have difficulty accessing supplementary content should contact the authors for assistance.

Ceftazidime/Avibactam Resistance in Carbapenemase-Producing *Klebsiella pneumoniae*

Appendix

Appendix Table 1. Antimicrobial susceptibility of KP0714 and the corresponding mutants isolated from a man with CAZ/AVI-resistant *Klebsiella pneumoniae* infection, Shanxi Province, China, 2021

		bla$_{SHV-12}$ promoters		MIC (μg/mL)										
Strain	KPC variants	P1	P2	CAZ/AVI	IMP	MEM	TZP	CAZ	FEP	ATM	AMK	LEV	TGC	POL
KP0714	KPC-2	+	+	32	≥64	≥64	≥128	≥64	≥64	≥64	≥64	≥8	1	0.5
KP0714 KPC S130A	KPC-2 S130A	+	+	32	0.5	2	≥128	≥64	≥64	≥64	≥64	≥8	1	0.5
KP0714 ΔP1	KPC-2	-	+	2	≥64	≥64	≥128	≥64	≥64	≥64	≥64	≥8	1	0.5
KP0714 ΔP2	KPC-2	+	-	1	≥64	≥64	≥128	≥64	≥64	≥64	≥64	≥8	1	0.5

*AMK, amikacin; ATM, aztreonam; CAZ, ceftazidime; FEP, cefepime; IPM, imipenem; LVX, levofloxacin; MEM, meropenem; POL, polymycin B; TGC, tigecycline; TZP, piperacillin-tazobactam.

Appendix Table 2. Genomic characteristics of *Klebsiella pneumoniae* KP0714 isolated from a man with CAZ/AVI-resistant *Klebsiella pneumoniae* infection, Shanxi Province, China, 2021*

Characteristic	Size (bp)	Replicon type	Antimicrobial resistance genes	Virulence genes
Chromosome	5,440,573	NA	*bla*$_{SHV-12}$, *bla*$_{LAP-2}$, *tet(A)*, *qnrS1*, *sul1*, *dfrA1*, *aph(3')-Ia*, *aac(3)-IId*, *mph(A)*	*mrkABCDFHIJ* (fimbria type 3), *fimABCDEFGHIK* (fimbria type 1), *ybt 9*-ICEKp3 (yersiniabactin siderophore)
pVir-KP0714	254,123	IncFIB(K)	*bla*$_{OXA-1}$, *aac(6')-Ib-cr*, *catB3*, *arr3*, *sul1*	*iucABCDiutA* (aerobactin), *rmpA2* (regulator of mucoid phenotype)
pKPC-KP0714	74,021	IncFII(pHN7A8)	*bla*$_{KPC-2}$, *bla*$_{TEM-1}$, *rmtB*, *fosA3*	NA
p3-KP0714	10,060	ColRNAI	NA	NA
p4-KP0714	5,596	NA	NA	NA

*NA, not applicable.

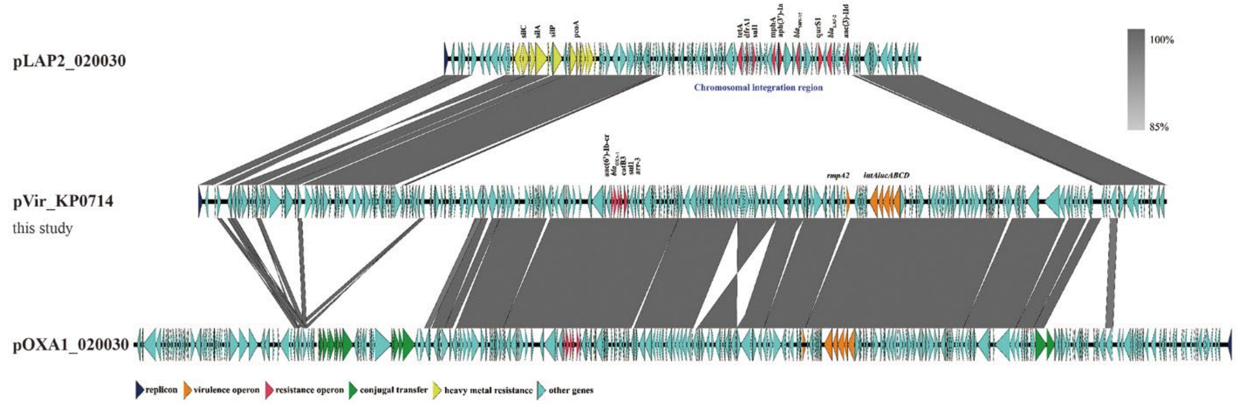

Appendix Figure 1. Linear alignment of plasmid pVir-KP0714 and virulence potential determination of *Klebsiella. pneumoniae* KP0714. Sequence alignment of plasmid pVir-KP0714 with pLAP2_020030 (GenBank accession no. CP028792) and pOXA1_020030 (GenBank accession no. CP028791). Arrows indicate genes, and colors are based on their functional classification.

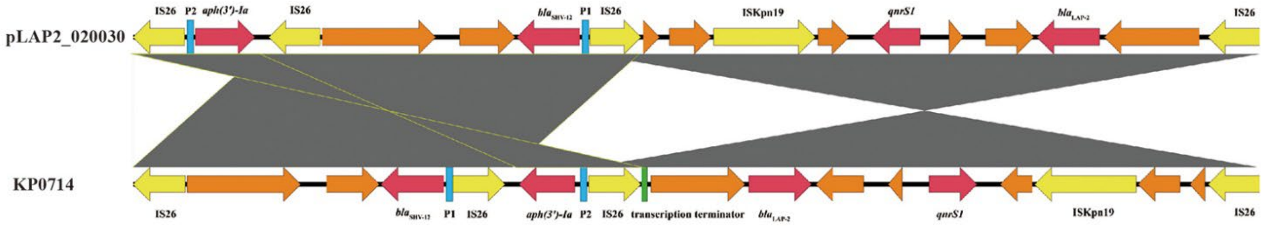

Appendix Figure 2. Overexpression of *bla*$_{\text{SHV-12}}$ contributing to ceftazidime/avibactam resistance in KP0714. Linear alignment of the 15-kb chromosomal integration genetic context containing *bla*$_{\text{SHV-12}}$ with pLAP2_020030.

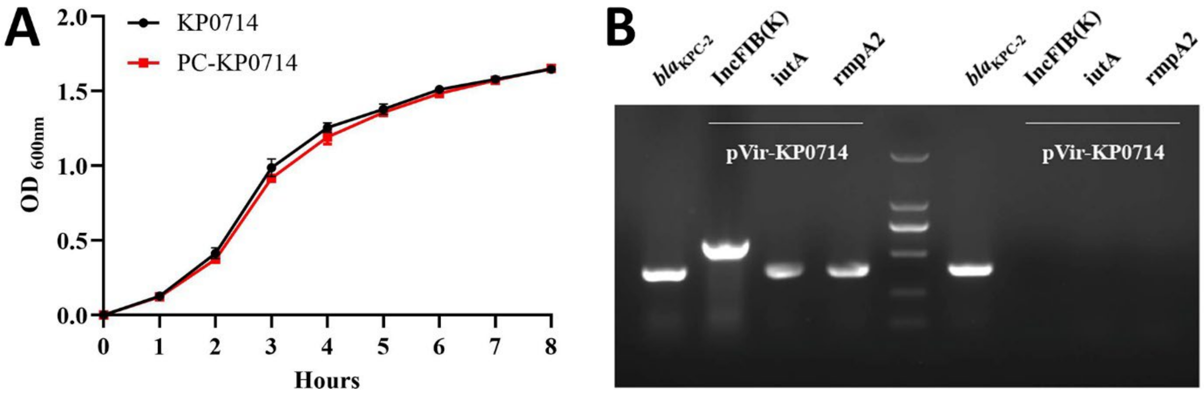

Appendix Figure 3. Plasmid curing of pVir-KP0714 in *K. pneumoniae* KP0714. A) Growth curves of KP0714 and pVir-KP0714-curing mutant PC-KP0714. B) PCR screening to identify the plasmid-curing mutant.
